# Severe Hyponatremia Caused by Sertraline-Induced Syndrome of Inappropriate Antidiuretic Hormone Secretion: A Complication With Critical Implications for Patient Safety

**DOI:** 10.7759/cureus.71309

**Published:** 2024-10-12

**Authors:** Sheldon W Joseph, Krista E Grant, John M Sousou, Nadim Qadir, Pramod Reddy

**Affiliations:** 1 Internal Medicine, University of Florida College of Medicine, Gainesville, USA; 2 Internal Medicine, University of Florida College of Medicine–Jacksonville, Jacksonville, USA

**Keywords:** hyponatremia, psychiatry, selective serotonin reuptake inhibitor (ssri), sodium, syndrome of inappropriate secretion of antidiuretic hormone (siadh)

## Abstract

Selective serotonin reuptake inhibitors (SSRIs) are commonly prescribed to treat various mental health conditions, including anxiety, depression, obsessive-compulsive disorder, and post-traumatic stress disorder. While these medications are generally effective, they can occasionally cause rare but serious side effects, such as hyponatremia, which often results from the syndrome of inappropriate antidiuretic hormone secretion (SIADH). Although hyponatremia is uncommon, severe cases can have critical consequences for patient safety. This case report describes a 68-year-old woman who developed severe hyponatremia after starting sertraline. Given the risk of severe hyponatremia, healthcare providers should exercise caution when prescribing SSRIs, considering the patient's comorbidities and the potential need for regular electrolyte monitoring. This case underscores the importance of vigilant monitoring for adverse effects and adjusting treatment strategies to ensure patient safety during psychiatric care.

## Introduction

Selective serotonin reuptake inhibitors (SSRIs) are a widely used class of antidepressants prescribed for various mental health conditions, including depression, anxiety disorders, obsessive-compulsive disorder, panic disorder, and post-traumatic stress disorder. They are often the first-line treatment for depression due to their efficacy and relatively fewer side effects compared to other antidepressants. SSRIs function by blocking the reuptake of serotonin in the brain, thereby increasing serotonin levels to improve mood and regulate other functions affected in several psychiatric disorders.

A significant potential side effect of SSRIs is hyponatremia, with incidence rates ranging from 0.5% to 32% [1]. Major risk factors for developing hyponatremia secondary to SSRI use include older age, female gender, concomitant use of diuretics, low body weight, and lower baseline serum sodium concentration [1]. The underlying mechanism for SSRI-induced hyponatremia is typically the syndrome of inappropriate antidiuretic hormone secretion (SIADH), where the body retains water excessively due to abnormal antidiuretic hormone secretion, leading to dilutional hyponatremia [2]. This condition results in euvolemic hyponatremia, where the body's total water content is normal, but the sodium concentration is low. In some cases, hyponatremia can persist even after discontinuation of the offending SSRI, necessitating careful monitoring and management.

We present the case of an elderly patient who developed severe hyponatremia following the recent initiation of sertraline. This case highlights the importance of recognizing that while medications such as SSRIs are widely used in clinical practice, clinicians must remain vigilant for potentially serious side effects.

## Case presentation

A 68-year-old female presented to the emergency department following a fall at her assisted living facility. Her medical history is significant for hypertension, hyperlipidemia, type 2 diabetes mellitus, and schizoaffective disorder. Her home medications include amlodipine 5 mg daily, risperidone 1 mg twice daily, and sertraline 100 mg daily, which she began approximately one month prior to presentation. Upon admission to the hospital, the patient reportedly exhibited symptoms of dizziness, lightheadedness, and nausea preceding the fall, along with a loss of consciousness. Laboratory evaluation revealed severe hyponatremia, with a sodium level of 104 mmol/L and a low serum osmolality of 224.1, consistent with hypovolemic hyponatremia. Other notable lab values included a chloride level of 69 mmol/L and a bicarbonate level of 18 mmol/L, among other tests (Table 1).

**Table 1:** Patient’s notable laboratory values on admission

Laboratory Test (Serum)	Patient’s Value	Reference Range
Sodium	104 mmol/L	135–145 mmol/L
Potassium	3.6 mmol/L	3.3–4.6 mmol/L
Chloride	69 mmol/L	101–110 mmol/L
Bicarbonate	18 mmol/L	21–29 mmol/L
Serum osmolality	224.1	275–295
Glucose	107 mg/dL	71–99 mg/dL
Cortisol (AM)	21.3 mcg/dL	5–23 mcg/dL
Thyroid-stimulating hormone	1.86 mU/L	0.27–4.20 mU/L

**Figure 1:**
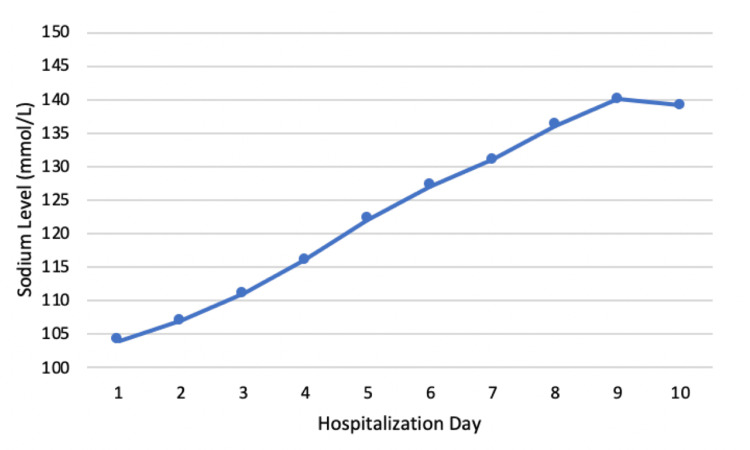
Patient’s daily sodium level throughout hospitalization

A repeat basic metabolic panel confirmed persistent hyponatremia, prompting the administration of intravenous hypertonic saline (3%). Urine chemistry revealed a urine sodium level of 152 mmol/L and urine osmolality of 423 mOsm/kg. Imaging studies, including a chest X-ray and computed tomography scans of the head and spine, were unremarkable. The patient was subsequently admitted to the medical intensive care unit for further management of symptomatic severe hyponatremia. Her sodium levels gradually improved with a 1 L/day fluid restriction, and the total daily correction did not exceed 8 mmol/L (Figure 1). Sertraline was ultimately discontinued from her home medications and listed as a contraindication due to severe hyponatremia. The patient was discharged several days later after her sodium levels had normalized, without any further complications.

## Discussion

The patient's decreased serum sodium levels, low serum osmolality, and elevated urine sodium are consistent with SIADH. SIADH secondary to sertraline use is relatively uncommon, with an incidence of approximately 11.7% among reported cases of SSRI-induced hyponatremia [3]. The significant complications of SIADH stem from the degree of hyponatremia. Severe hyponatremia, defined as a sodium level of less than 120 mEq/L, can lead to life-threatening neurological complications, including seizures, coma, and death. Untreated hyponatremia may also affect bone metabolism, leading to secondary osteoporosis with a significantly increased risk of gait instability, falls, and subsequent fractures [4]. Additionally, studies have demonstrated a high mortality rate and prolonged hospital admissions in patients with severe hyponatremia secondary to SIADH [5].

This case highlights the rare but possible adverse effect of severe hyponatremia due to SIADH in patients using sertraline or other SSRIs. There are documented cases in the medical literature of moderate hyponatremia secondary to sertraline use, with resolution in sodium levels following fluid restriction and discontinuation of the medication [6]. As SIADH is a recognized potential side effect of SSRIs, it is crucial to underscore the risk of severe hyponatremia and the potentially fatal complications that may accompany it. Early recognition of symptoms in the context of recent SSRI use and increased water intake can prompt timely investigation of the side effect profile of a patient’s home medications.

Healthcare providers should prioritize pharmacovigilance, monitor fluid balance carefully, and conduct individualized risk assessments when treating patients with SSRIs. It is also crucial to educate patients about the symptoms of hyponatremia and the need for prompt intervention. Currently, there are no specific guidelines recommending routine laboratory tests such as complete blood counts or BMPs for patients on SSRIs. However, clinicians should consider individual risk factors, such as pre-existing metabolic conditions or hematologic abnormalities, when determining the need for more frequent laboratory monitoring. Further research should explore the long-term effects of SSRI-induced hyponatremia, potential genetic factors that may increase susceptibility to this condition, and the role of polypharmacy in causing electrolyte imbalances.

## Conclusions

This case report highlights the significant risks of hyponatremia associated with SSRIs such as sertraline, as demonstrated in our patient. It emphasizes the need to consider these risks in psychiatric treatment and calls for a more attentive, multidisciplinary approach when prescribing SSRIs. While SSRIs are effective for various mental health conditions, healthcare providers should remain vigilant for the potential side effect of severe hyponatremia, particularly in high-risk populations. Understanding the risk factors, mechanisms, and management strategies for SSRI-induced hyponatremia is essential to ensure safe and effective prescribing practices.
